# *catena*-Poly[[(1,10-phenanthroline-κ^2^ *N*,*N*′)zinc]-μ-4-sulfonato­benzo­triazolido-κ^3^ *N* ^3^,*O*:*N* ^1^]

**DOI:** 10.1107/S160053681104743X

**Published:** 2011-11-12

**Authors:** Xiao-Chun Cheng

**Affiliations:** a Faculty of Life Science and Chemical Engineering, Huaiyin Institute of Technology, Huaian 223003, People’s Republic of China

## Abstract

In the title complex, [Zn(C_6_H_3_N_3_O_3_S)(C_12_H_8_N_2_)]_*n*_, the Zn^2+^ cation is coordinated by two N atoms from two 4-sulfonato­benzotriazolide dianions, two N atoms from a 1,10-phenanthroline mol­ecule and a sulfonate O atom from a 4-sulfonato­benzotriazolide anion, displaying a distorted ZnN_4_O trigonal–bipyramidal geometry. Each 1,10-phenanthroline ligand displays a bidentate chelating coordinating mode and the 4-sulfonato­benzotriazolide ions act as μ_2_-bridges, linking different Zn^2+^ cations into a chain along the *b* axis. The crystal structure is consolidated by C—H⋯O hydrogen-bonding inter­actions.

## Related literature

For related structures, see: Xia *et al.* (2010 ▶).
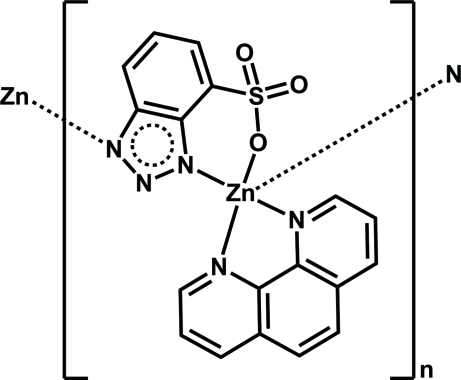


## Experimental

### 

#### Crystal data

[Zn(C_6_H_3_N_3_O_3_S)(C_12_H_8_N_2_)]
*M* *_r_* = 442.75
Orthorhombic, 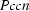

*a* = 14.5562 (19) Å
*b* = 25.903 (3) Å
*c* = 8.9239 (12) Å
*V* = 3364.8 (8) Å^3^
*Z* = 8
Mo *K*α radiation
μ = 1.62 mm^−1^
*T* = 293 K
0.20 × 0.20 × 0.20 mm

#### Data collection

Bruker SMART APEXII CCD diffractometer
Absorption correction: multi-scan (*SADABS*; Sheldrick, 1996 ▶) *T*_min_ = 0.738, *T*_max_ = 0.738
19596 measured reflections
3819 independent reflections
2895 reflections with *I* > 2σ(*I*)
*R*_int_ = 0.092

#### Refinement

*R*[*F* ^2^ > 2σ(*F* ^2^)] = 0.045
*wR*(*F* ^2^) = 0.147
*S* = 1.10
3819 reflections
253 parameters
H-atom parameters constrained
Δρ_max_ = 0.82 e Å^−3^
Δρ_min_ = −0.70 e Å^−3^

### 

Data collection: *APEX2* (Bruker, 2008 ▶); cell refinement: *SAINT* (Bruker, 2008 ▶); data reduction: *SAINT*; program(s) used to solve structure: *SHELXS97* (Sheldrick, 2008 ▶); program(s) used to refine structure: *SHELXL97* (Sheldrick, 2008 ▶); molecular graphics: *DIAMOND* (Brandenburg, 2000 ▶); software used to prepare material for publication: *SHELXTL* (Sheldrick, 2008 ▶).

## Supplementary Material

Crystal structure: contains datablock(s) I, global. DOI: 10.1107/S160053681104743X/pv2482sup1.cif

Structure factors: contains datablock(s) I. DOI: 10.1107/S160053681104743X/pv2482Isup2.hkl

Supplementary material file. DOI: 10.1107/S160053681104743X/pv2482Isup3.cdx

Additional supplementary materials:  crystallographic information; 3D view; checkCIF report

## Figures and Tables

**Table 1:** Hydrogen-bond geometry (Å, °)

*D*—H⋯*A*	*D*—H	H⋯*A*	*D*⋯*A*	*D*—H⋯*A*
C9—H9⋯O2^i^	0.93	2.49	3.351 (5)	154
C12—H12⋯O2^ii^	0.93	2.55	3.369 (5)	148

Symmetry codes: (i) 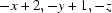; (ii) 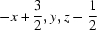.

**Fig. 1.**
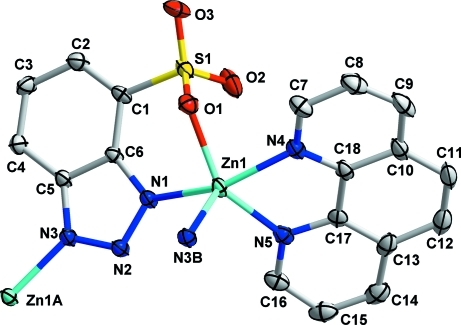
: The coordination environment of zinc ion in the title complex with the ellipsoids drawn at the 30% probability level. The hydrogen atoms are omitted for clarity. Symmetry codes: A = x, -y + 1/2, z + 1/2; B = x, -y + 1/2, z - 1/2.

## supplementary crystallographic information

### Comment

Benzotriazole-4-sulfonic acid is often used as a ligand to synthesize complexes for its variable coordination modes. Herein, we report the crystal structure of the title complex. The asymmetric unit consists of one zinc ion, one 1,10-phenanthroline molecule, and one 4-sulfonatobenzotriazolide anion. each Zn ion is coordinated by two N atoms from two different 4-sulfonatobenzotriazolide anions, two N atoms from one 1,10-phenanthroline molecule, and one sulfonate O atoms from one 4-sulfonatobenzotriazolide anions, displaying a distorted ZnN_4_O trigonal bipyramidal geometry (Fig. 1). Each 1,10-phenanthroline displays a bidentate coordinating mode. And every 4-sulfonatobenzotriazolide acts as a µ_2_-bridge, linking different zinc ions to form a one-dimensional chain along the *b* axis direction. The crystal structure is consolidated by hydrogen bonding interactions of the type C—H···O (Table 1).

### Experimental

A mixture of zinc nitrate hexahydrate (59.4 mg, 0.2 mmol), benzotriazole-4-sulfonic acid (39.8 mg, 0.2 mmol), 1,10-phenanthroline (36.0 mg, 0.2 mmol) and potassium hydroxide (22.4 mg, 0.4 mmol) in 12 ml H_2_O was sealed in a 16 ml Teflon-lined stainless steel container and heated to 413 K for 3 days. After cooling the container to the room temperature, colorless block crystals of the title complex were obtained.

### Refinement

The hydrogen atoms were located in geometrically idealized positions and constrained to ride on their parent atoms, with C—H = 0.93 Å and *U*_iso_(H) = 1.2*U*_eq_(C).

### Crystal data

**Table:**

[Zn(C_6_H_3_N_3_O_3_S)(C_12_H_8_N_2_)]	*F*(000) = 1792
*M**_r_* = 442.75	*D*_x_ = 1.748 Mg m^−^^3^
Orthorhombic, *P**c**c**n*	Mo *K*α radiation, λ = 0.71073 Å
Hall symbol: -P 2ab 2ac	Cell parameters from 2885 reflections
*a* = 14.5562 (19) Å	θ = 2.7–25.0°
*b* = 25.903 (3) Å	µ = 1.62 mm^−^^1^
*c* = 8.9239 (12) Å	*T* = 293 K
*V* = 3364.8 (8) Å^3^	Block, colorless
*Z* = 8	0.20 × 0.20 × 0.20 mm

### Data collection

**Table:**

Bruker SMART APEXII CCD diffractometer	3819 independent reflections
Radiation source: fine-focus sealed tube	2895 reflections with *I* > 2σ(*I*)
graphite	*R*_int_ = 0.092
φ and ω scans	θ_max_ = 27.4°, θ_min_ = 1.6°
Absorption correction: multi-scan (*SADABS*; Sheldrick, 1996)	*h* = −18→18
*T*_min_ = 0.738, *T*_max_ = 0.738	*k* = −33→33
19596 measured reflections	*l* = −7→11

### Refinement

**Table:**

Refinement on *F*^2^	Primary atom site location: structure-invariant direct methods
Least-squares matrix: full	Secondary atom site location: difference Fourier map
*R*[*F*^2^ > 2σ(*F*^2^)] = 0.045	Hydrogen site location: inferred from neighbouring sites
*wR*(*F*^2^) = 0.147	H-atom parameters constrained
*S* = 1.10	*w* = 1/[σ^2^(*F*_o_^2^) + (0.0628*P*)^2^ + 1.2933*P*] where *P* = (*F*_o_^2^ + 2*F*_c_^2^)/3
3819 reflections	(Δ/σ)_max_ = 0.001
253 parameters	Δρ_max_ = 0.82 e Å^−^^3^
0 restraints	Δρ_min_ = −0.70 e Å^−^^3^

### Special details

**Table:**

Geometry. All e.s.d.'s (except the e.s.d. in the dihedral angle between two l.s. planes) are estimated using the full covariance matrix. The cell e.s.d.'s are taken into account individually in the estimation of e.s.d.'s in distances, angles and torsion angles; correlations between e.s.d.'s in cell parameters are only used when they are defined by crystal symmetry. An approximate (isotropic) treatment of cell e.s.d.'s is used for estimating e.s.d.'s involving l.s. planes.
Refinement. Refinement of *F*^2^ against ALL reflections. The weighted *R*-factor *wR* and goodness of fit *S* are based on *F*^2^, conventional *R*-factors *R* are based on *F*, with *F* set to zero for negative *F*^2^. The threshold expression of *F*^2^ > σ(*F*^2^) is used only for calculating *R*-factors(gt) *etc*. and is not relevant to the choice of reflections for refinement. *R*-factors based on *F*^2^ are statistically about twice as large as those based on *F*, and *R*- factors based on ALL data will be even larger.

### Fractional atomic coordinates and isotropic or equivalent isotropic displacement parameters (Å^2^)

**Table:**

	*x*	*y*	*z*	*U*_iso_*/*U*_eq_	
Zn1	0.97927 (3)	0.330127 (13)	0.19495 (5)	0.02895 (17)	
N5	0.8384 (2)	0.34450 (11)	0.1826 (3)	0.0334 (7)	
C17	0.8148 (2)	0.38273 (12)	0.0857 (4)	0.0333 (8)	
C9	0.9411 (4)	0.47031 (16)	−0.1724 (5)	0.0581 (13)	
H9	0.9313	0.4973	−0.2392	0.070*	
C10	0.8673 (3)	0.44934 (14)	−0.0912 (5)	0.0454 (10)	
C13	0.7232 (3)	0.39803 (14)	0.0600 (5)	0.0421 (9)	
C14	0.6559 (3)	0.36901 (16)	0.1331 (5)	0.0497 (11)	
H14	0.5942	0.3767	0.1170	0.060*	
C7	1.0419 (3)	0.41028 (15)	−0.0526 (5)	0.0459 (10)	
H7	1.1012	0.3977	−0.0398	0.055*	
C18	0.8878 (3)	0.40801 (12)	0.0054 (4)	0.0337 (8)	
C16	0.7712 (3)	0.31927 (15)	0.2529 (5)	0.0420 (9)	
H16	0.7865	0.2937	0.3216	0.050*	
C15	0.6784 (3)	0.32995 (16)	0.2270 (6)	0.0508 (12)	
H15	0.6328	0.3105	0.2735	0.061*	
N4	0.9732 (2)	0.38901 (11)	0.0256 (4)	0.0356 (7)	
S1	1.10686 (6)	0.40039 (3)	0.37277 (12)	0.0366 (2)	
N1	0.99027 (19)	0.29840 (10)	0.4183 (3)	0.0279 (6)	
N2	0.9579 (2)	0.25262 (10)	0.4630 (3)	0.0312 (6)	
C1	1.1157 (2)	0.35655 (11)	0.5213 (4)	0.0293 (7)	
C2	1.1777 (3)	0.35925 (14)	0.6343 (5)	0.0392 (9)	
H2	1.2171	0.3875	0.6391	0.047*	
C3	1.1841 (3)	0.32053 (16)	0.7447 (5)	0.0441 (10)	
H3	1.2267	0.3242	0.8217	0.053*	
O1	1.10445 (18)	0.36563 (10)	0.2416 (3)	0.0391 (6)	
O3	1.1878 (2)	0.43226 (10)	0.3728 (4)	0.0511 (8)	
O2	1.0199 (2)	0.42648 (11)	0.3896 (4)	0.0628 (10)	
N3	1.0018 (2)	0.23706 (11)	0.5874 (3)	0.0307 (6)	
C6	1.0567 (2)	0.31302 (11)	0.5174 (4)	0.0257 (7)	
C5	1.0640 (2)	0.27433 (12)	0.6254 (4)	0.0294 (7)	
C4	1.1290 (3)	0.27760 (15)	0.7414 (4)	0.0375 (8)	
H4	1.1344	0.2517	0.8130	0.045*	
C12	0.7058 (3)	0.44058 (16)	−0.0377 (6)	0.0551 (12)	
H12	0.6456	0.4514	−0.0526	0.066*	
C11	0.7738 (3)	0.46542 (16)	−0.1083 (6)	0.0581 (13)	
H11	0.7602	0.4935	−0.1692	0.070*	
C8	1.0273 (3)	0.45068 (18)	−0.1525 (6)	0.0553 (12)	
H8	1.0764	0.4644	−0.2059	0.066*	

### Atomic displacement parameters (Å^2^)

**Table:**

	*U*^11^	*U*^22^	*U*^33^	*U*^12^	*U*^13^	*U*^23^
Zn1	0.0395 (3)	0.0203 (2)	0.0271 (3)	0.00316 (14)	−0.00219 (17)	−0.00023 (15)
N5	0.0423 (17)	0.0270 (14)	0.0309 (17)	−0.0015 (12)	−0.0001 (13)	−0.0032 (12)
C17	0.0427 (19)	0.0239 (16)	0.033 (2)	0.0031 (13)	−0.0079 (16)	−0.0106 (14)
C9	0.100 (4)	0.032 (2)	0.043 (3)	−0.005 (2)	−0.005 (3)	0.0132 (18)
C10	0.074 (3)	0.0236 (17)	0.038 (2)	0.0022 (17)	−0.016 (2)	0.0010 (16)
C13	0.045 (2)	0.041 (2)	0.041 (2)	0.0082 (17)	−0.0131 (19)	−0.0133 (17)
C14	0.041 (2)	0.052 (2)	0.055 (3)	0.0053 (18)	−0.006 (2)	−0.022 (2)
C7	0.054 (2)	0.036 (2)	0.048 (3)	−0.0056 (17)	0.007 (2)	0.0085 (18)
C18	0.048 (2)	0.0231 (16)	0.0296 (19)	0.0012 (14)	−0.0052 (16)	−0.0027 (14)
C16	0.051 (2)	0.0347 (19)	0.041 (2)	−0.0063 (17)	0.007 (2)	−0.0041 (17)
C15	0.046 (2)	0.047 (3)	0.059 (3)	−0.0087 (18)	0.008 (2)	−0.018 (2)
N4	0.0468 (18)	0.0287 (15)	0.0312 (17)	0.0009 (12)	−0.0005 (14)	0.0031 (13)
S1	0.0427 (5)	0.0234 (4)	0.0437 (6)	−0.0010 (3)	0.0040 (4)	0.0088 (4)
N1	0.0386 (15)	0.0217 (13)	0.0232 (14)	0.0007 (11)	−0.0012 (12)	0.0012 (11)
N2	0.0422 (16)	0.0241 (14)	0.0273 (15)	−0.0041 (11)	−0.0026 (13)	0.0017 (12)
C1	0.0381 (18)	0.0188 (15)	0.0311 (18)	−0.0014 (12)	0.0053 (15)	−0.0012 (13)
C2	0.045 (2)	0.0339 (19)	0.038 (2)	−0.0103 (15)	−0.0016 (18)	−0.0019 (16)
C3	0.045 (2)	0.051 (2)	0.036 (2)	−0.0136 (18)	−0.016 (2)	0.0041 (19)
O1	0.0430 (14)	0.0394 (15)	0.0350 (14)	−0.0068 (11)	0.0000 (12)	0.0070 (12)
O3	0.0644 (18)	0.0334 (14)	0.0557 (19)	−0.0201 (13)	0.0057 (15)	0.0051 (13)
O2	0.060 (2)	0.0438 (17)	0.084 (3)	0.0201 (14)	0.0185 (17)	0.0227 (17)
N3	0.0406 (16)	0.0248 (14)	0.0267 (16)	−0.0011 (11)	−0.0032 (13)	0.0029 (12)
C6	0.0327 (17)	0.0222 (14)	0.0221 (16)	−0.0027 (12)	0.0009 (14)	−0.0006 (12)
C5	0.0370 (18)	0.0223 (15)	0.0288 (18)	−0.0012 (13)	−0.0008 (15)	0.0001 (13)
C4	0.042 (2)	0.038 (2)	0.032 (2)	−0.0055 (15)	−0.0067 (17)	0.0088 (16)
C12	0.059 (3)	0.051 (3)	0.056 (3)	0.021 (2)	−0.025 (2)	−0.011 (2)
C11	0.078 (3)	0.035 (2)	0.061 (3)	0.015 (2)	−0.028 (3)	0.006 (2)
C8	0.073 (3)	0.046 (2)	0.048 (3)	−0.014 (2)	0.006 (2)	0.010 (2)

### Geometric parameters (Å, °)

**Table:**

Zn1—N3^i^	2.014 (3)	C15—H15	0.9300
Zn1—O1	2.083 (3)	S1—O3	1.439 (3)
Zn1—N5	2.086 (3)	S1—O2	1.443 (3)
Zn1—N4	2.149 (3)	S1—O1	1.477 (3)
Zn1—N1	2.162 (3)	S1—C1	1.750 (4)
N5—C16	1.334 (5)	N1—N2	1.337 (4)
N5—C17	1.359 (5)	N1—C6	1.364 (4)
C17—C13	1.410 (5)	N2—N3	1.343 (4)
C17—C18	1.440 (5)	C1—C2	1.355 (5)
C9—C8	1.365 (7)	C1—C6	1.418 (4)
C9—C10	1.405 (7)	C2—C3	1.409 (6)
C9—H9	0.9300	C2—H2	0.9300
C10—C18	1.407 (5)	C3—C4	1.372 (5)
C10—C11	1.431 (6)	C3—H3	0.9300
C13—C14	1.397 (6)	N3—C5	1.366 (4)
C13—C12	1.427 (6)	N3—Zn1^ii^	2.014 (3)
C14—C15	1.354 (7)	C6—C5	1.395 (4)
C14—H14	0.9300	C5—C4	1.405 (5)
C7—N4	1.338 (5)	C4—H4	0.9300
C7—C8	1.391 (6)	C12—C11	1.338 (7)
C7—H7	0.9300	C12—H12	0.9300
C18—N4	1.349 (5)	C11—H11	0.9300
C16—C15	1.398 (6)	C8—H8	0.9300
C16—H16	0.9300		
			
N3^i^—Zn1—O1	109.52 (12)	C18—N4—Zn1	113.0 (2)
N3^i^—Zn1—N5	106.80 (12)	O3—S1—O2	116.73 (18)
O1—Zn1—N5	142.30 (11)	O3—S1—O1	111.69 (17)
N3^i^—Zn1—N4	106.53 (12)	O2—S1—O1	110.3 (2)
O1—Zn1—N4	82.14 (11)	O3—S1—C1	108.17 (18)
N5—Zn1—N4	78.21 (11)	O2—S1—C1	106.83 (18)
N3^i^—Zn1—N1	95.67 (11)	O1—S1—C1	101.91 (15)
O1—Zn1—N1	85.34 (11)	N2—N1—C6	107.6 (3)
N5—Zn1—N1	100.92 (11)	N2—N1—Zn1	125.9 (2)
N4—Zn1—N1	157.12 (11)	C6—N1—Zn1	123.0 (2)
C16—N5—C17	118.1 (3)	N1—N2—N3	110.2 (3)
C16—N5—Zn1	127.5 (3)	C2—C1—C6	117.6 (3)
C17—N5—Zn1	114.4 (2)	C2—C1—S1	125.4 (3)
N5—C17—C13	123.2 (4)	C6—C1—S1	116.9 (3)
N5—C17—C18	117.5 (3)	C1—C2—C3	121.9 (3)
C13—C17—C18	119.3 (3)	C1—C2—H2	119.1
C8—C9—C10	119.4 (4)	C3—C2—H2	119.1
C8—C9—H9	120.3	C4—C3—C2	121.5 (4)
C10—C9—H9	120.3	C4—C3—H3	119.2
C9—C10—C18	116.6 (4)	C2—C3—H3	119.2
C9—C10—C11	124.0 (4)	S1—O1—Zn1	116.64 (16)
C18—C10—C11	119.3 (4)	N2—N3—C5	108.0 (3)
C14—C13—C17	115.8 (4)	N2—N3—Zn1^ii^	125.1 (2)
C14—C13—C12	125.3 (4)	C5—N3—Zn1^ii^	126.9 (2)
C17—C13—C12	118.9 (4)	N1—C6—C5	107.6 (3)
C15—C14—C13	121.4 (4)	N1—C6—C1	131.8 (3)
C15—C14—H14	119.3	C5—C6—C1	120.5 (3)
C13—C14—H14	119.3	N3—C5—C6	106.6 (3)
N4—C7—C8	122.0 (4)	N3—C5—C4	132.2 (3)
N4—C7—H7	119.0	C6—C5—C4	121.1 (3)
C8—C7—H7	119.0	C3—C4—C5	117.3 (3)
N4—C18—C10	123.7 (4)	C3—C4—H4	121.3
N4—C18—C17	116.6 (3)	C5—C4—H4	121.3
C10—C18—C17	119.6 (3)	C11—C12—C13	121.9 (4)
N5—C16—C15	122.3 (4)	C11—C12—H12	119.1
N5—C16—H16	118.9	C13—C12—H12	119.1
C15—C16—H16	118.9	C12—C11—C10	120.9 (4)
C14—C15—C16	119.0 (4)	C12—C11—H11	119.6
C14—C15—H15	120.5	C10—C11—H11	119.6
C16—C15—H15	120.5	C9—C8—C7	120.3 (4)
C7—N4—C18	117.9 (3)	C9—C8—H8	119.9
C7—N4—Zn1	128.9 (3)	C7—C8—H8	119.9
			
N3^i^—Zn1—N5—C16	70.4 (3)	N3^i^—Zn1—N1—C6	117.1 (3)
O1—Zn1—N5—C16	−125.6 (3)	O1—Zn1—N1—C6	7.9 (3)
N4—Zn1—N5—C16	174.3 (3)	N5—Zn1—N1—C6	−134.6 (3)
N1—Zn1—N5—C16	−29.0 (3)	N4—Zn1—N1—C6	−49.0 (4)
N3^i^—Zn1—N5—C17	−106.6 (2)	C6—N1—N2—N3	−0.4 (4)
O1—Zn1—N5—C17	57.5 (3)	Zn1—N1—N2—N3	158.8 (2)
N4—Zn1—N5—C17	−2.6 (2)	O3—S1—C1—C2	12.6 (4)
N1—Zn1—N5—C17	154.0 (2)	O2—S1—C1—C2	−113.8 (4)
C16—N5—C17—C13	2.1 (5)	O1—S1—C1—C2	130.4 (3)
Zn1—N5—C17—C13	179.4 (3)	O3—S1—C1—C6	−163.3 (3)
C16—N5—C17—C18	−177.2 (3)	O2—S1—C1—C6	70.3 (3)
Zn1—N5—C17—C18	0.0 (4)	O1—S1—C1—C6	−45.5 (3)
C8—C9—C10—C18	−1.1 (6)	C6—C1—C2—C3	0.0 (6)
C8—C9—C10—C11	−177.1 (4)	S1—C1—C2—C3	−175.9 (3)
N5—C17—C13—C14	−4.2 (5)	C1—C2—C3—C4	1.3 (7)
C18—C17—C13—C14	175.2 (3)	O3—S1—O1—Zn1	−165.49 (16)
N5—C17—C13—C12	176.5 (3)	O2—S1—O1—Zn1	−33.9 (2)
C18—C17—C13—C12	−4.2 (5)	C1—S1—O1—Zn1	79.25 (19)
C17—C13—C14—C15	2.3 (6)	N3^i^—Zn1—O1—S1	−152.98 (16)
C12—C13—C14—C15	−178.3 (4)	N5—Zn1—O1—S1	43.2 (3)
C9—C10—C18—N4	1.6 (6)	N4—Zn1—O1—S1	102.19 (18)
C11—C10—C18—N4	177.9 (4)	N1—Zn1—O1—S1	−58.61 (17)
C9—C10—C18—C17	−176.5 (3)	N1—N2—N3—C5	0.7 (4)
C11—C10—C18—C17	−0.2 (5)	N1—N2—N3—Zn1^ii^	−178.7 (2)
N5—C17—C18—N4	4.5 (5)	N2—N1—C6—C5	0.0 (4)
C13—C17—C18—N4	−174.8 (3)	Zn1—N1—C6—C5	−160.0 (2)
N5—C17—C18—C10	−177.2 (3)	N2—N1—C6—C1	177.1 (3)
C13—C17—C18—C10	3.4 (5)	Zn1—N1—C6—C1	17.1 (5)
C17—N5—C16—C15	1.8 (6)	C2—C1—C6—N1	−177.8 (4)
Zn1—N5—C16—C15	−175.0 (3)	S1—C1—C6—N1	−1.6 (5)
C13—C14—C15—C16	1.2 (7)	C2—C1—C6—C5	−1.0 (5)
N5—C16—C15—C14	−3.5 (7)	S1—C1—C6—C5	175.3 (3)
C8—C7—N4—C18	−0.2 (6)	N2—N3—C5—C6	−0.7 (4)
C8—C7—N4—Zn1	−175.8 (3)	Zn1^ii^—N3—C5—C6	178.7 (2)
C10—C18—N4—C7	−1.0 (5)	N2—N3—C5—C4	−178.1 (4)
C17—C18—N4—C7	177.2 (3)	Zn1^ii^—N3—C5—C4	1.3 (6)
C10—C18—N4—Zn1	175.3 (3)	N1—C6—C5—N3	0.5 (4)
C17—C18—N4—Zn1	−6.6 (4)	C1—C6—C5—N3	−177.1 (3)
N3^i^—Zn1—N4—C7	−75.0 (3)	N1—C6—C5—C4	178.2 (3)
O1—Zn1—N4—C7	33.1 (3)	C1—C6—C5—C4	0.6 (5)
N5—Zn1—N4—C7	−179.2 (4)	C2—C3—C4—C5	−1.6 (6)
N1—Zn1—N4—C7	90.6 (4)	N3—C5—C4—C3	177.7 (4)
N3^i^—Zn1—N4—C18	109.2 (3)	C6—C5—C4—C3	0.7 (6)
O1—Zn1—N4—C18	−142.6 (3)	C14—C13—C12—C11	−177.5 (4)
N5—Zn1—N4—C18	5.0 (2)	C17—C13—C12—C11	1.8 (6)
N1—Zn1—N4—C18	−85.2 (4)	C13—C12—C11—C10	1.5 (7)
N3^i^—Zn1—N1—N2	−39.2 (3)	C9—C10—C11—C12	173.7 (5)
O1—Zn1—N1—N2	−148.4 (3)	C18—C10—C11—C12	−2.3 (6)
N5—Zn1—N1—N2	69.1 (3)	C10—C9—C8—C7	0.0 (7)
N4—Zn1—N1—N2	154.7 (3)	N4—C7—C8—C9	0.7 (7)

Symmetry codes: (i) *x*, −*y*+1/2, *z*−1/2; (ii) *x*, −*y*+1/2, *z*+1/2.

### Hydrogen-bond geometry (Å, °)

**Table:**

*D*—H···*A*	*D*—H	H···*A*	*D*···*A*	*D*—H···*A*
C9—H9···O2^iii^	0.93	2.49	3.351 (5)	154
C12—H12···O2^iv^	0.93	2.55	3.369 (5)	148

Symmetry codes: (iii) −*x*+2, −*y*+1, −*z*; (iv) −*x*+3/2, *y*, *z*−1/2.
